# An atypical presentation of Wellens’ syndrome with critical right coronary artery stenosis instead of left anterior descending artery stenosis: a case report

**DOI:** 10.1093/ehjcr/ytaf333

**Published:** 2025-07-14

**Authors:** Can Baba Arın, Mohamed Omar Hassan, Ishak Ahmed Abdi, Said Abdirahman Ahmed, Ahmed Elmi Abdi

**Affiliations:** Departments of Cardiology, Mogadishu Somali Turkey Recep Tayyip Erdoğan Training and Research Hospital, KM4 Square, Hodan District, Mogadishu 252, Somalia; Departments of Cardiology, Mogadishu Somali Turkey Recep Tayyip Erdoğan Training and Research Hospital, KM4 Square, Hodan District, Mogadishu 252, Somalia; Departments of Cardiology, Mogadishu Somali Turkey Recep Tayyip Erdoğan Training and Research Hospital, KM4 Square, Hodan District, Mogadishu 252, Somalia; Departments of Cardiology, Mogadishu Somali Turkey Recep Tayyip Erdoğan Training and Research Hospital, KM4 Square, Hodan District, Mogadishu 252, Somalia; Departments of Cardiology, Mogadishu Somali Turkey Recep Tayyip Erdoğan Training and Research Hospital, KM4 Square, Hodan District, Mogadishu 252, Somalia

**Keywords:** Case report, Wellens’ syndrome, Right coronary artery, Left anterior descending artery, Electrocardiogram, Biphasic T waves, Myocardial infarction

## Abstract

**Background:**

Wellens’ syndrome is identified by specific electrocardiographic changes biphasic or deeply inverted T waves in the precordial leads that are strongly associated with significant stenosis of the left anterior descending artery (LAD). This syndrome is regarded as a high-risk indicator of impending anterior wall myocardial infarction. While the classic association is with LAD stenosis, atypical presentations involving other coronary arteries, such as the right coronary artery (RCA), are uncommon but significant.

**Case summary:**

A 71-year-old male presented with exertional chest pain radiating to his back, which was relieved by rest. The patient had hypertension for 15 years and type 2 diabetes, which has been poorly controlled for 6 years. He also had a long history of using Khat, which may have contributed to his cardiovascular risk profile. Biphasic T waves in leads V2–V6 were detected on initial electrocardiography, which was consistent with Wellens’ syndrome Type A.

Although Wellens’ syndrome is classically associated with LAD stenosis, coronary angiography revealed a significant occlusion of the RCA rather than the LAD. The patient underwent successful percutaneous coronary intervention, requiring the implantation of two drug-eluting stents in the proximal and distal RCA. He showed significant clinical improvement following the procedure.

**Discussion:**

This case demonstrates an unusual and unexpected presentation of Wellens’ syndrome, in which the classic electrocardiographic findings of LAD ischaemia were mistaken for RCA involvement. The case emphasizes the significance of a thorough and comprehensive cardiac evaluation, as electrocardiographic findings do always correlate with the underlying coronary anatomy with rare exceptions. Although Wellens’ syndrome is most commonly associated with proximal LAD stenosis, RCA occlusion can cause similar ischaemic changes. This case serves as a reminder that in Wellens’ syndrome patients, alternative coronary pathologies should be considered, and prompt coronary angiography is critical for accurate diagnosis and optimal management.

Learning points

- Unusual coronary territory involvement: This case demonstrates a rare presentation of Wellens’ syndrome associated with critical stenosis in the right coronary artery, rather than the typically involved proximal left anterior descending artery.
- Diagnostic and clinical implications: The report underscores the importance of coronary angiography in patients with Wellens’-like electrocardiogram changes, as it can reveal unexpected coronary involvement and guide appropriate revascularization strategies.

## Introduction

Wellens’ syndrome is a clinical syndrome with a specific pattern of electrocardiogram (ECG) characterized by biphasic or deeply inverted T waves seen in precordial leads in V1–V6, which can indicate critical blockage or stenosis in one or more coronary arteries. Since then, Wellens’ syndrome gained recognition as a diagnostic tool to identify high-risk patients with acute coronary syndrome (ACS).^1^

Wellens’ syndrome is a distinct electrocardiographic (ECG) pattern characterized by biphasic or deeply inverted T waves in precordial leads (V1–V6), typically associated with critical stenosis of the proximal left anterior descending artery (LAD). First described by de Zwaan *et al*. in 1982, it serves as an important marker of impending myocardial infarction (MI) in high-risk patients with ACS.^1^

The syndrome is linked to the pathophysiological cycle of coronary stenosis, reperfusion, and restenosis, often presenting during a pain-free period. Diagnostic criteria include biphasic or deeply inverted T waves (V2–V5), minimal ST-elevation (V2–V3), normal R-wave progression, and mildly elevated cardiac markers.^2^

Wellens’ syndrome accounts for 15% of unstable angina cases, and prompt coronary intervention is crucial to prevent MI.^3^

Here, we present a rare case of Wellens’ syndrome in a male patient with ECG changes revealing biphasic T waves in leads V2–V6, which is characteristic of Wellens’ syndrome Type A, which is typically associated with proximal LAD stenosis; however, in this case, critical stenosis was found in the right coronary artery (RCA).

## Summary figure

**Figure:**
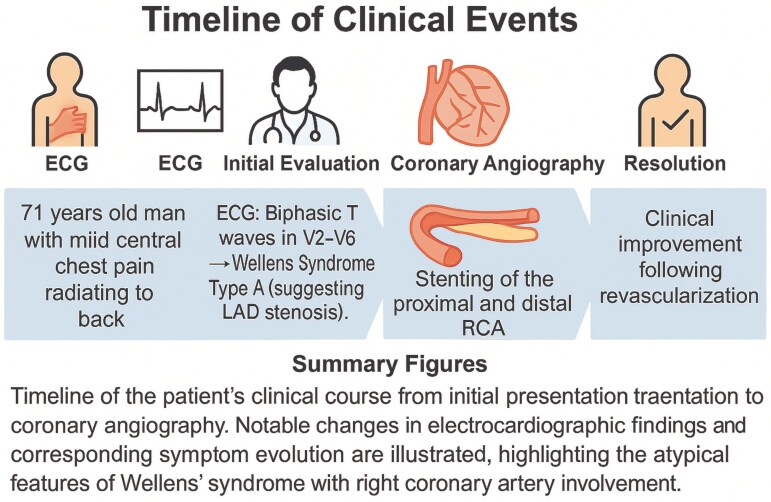


## Case presentation

A 71-year-old man presented to the emergency department with mild central chest pain that spread to his back. He had a 15-year history of hypertension treated with valsartan 80 mg daily as well as 6-year history of diabetes mellitus poor medication adherence. The patient also had a long history of Khat use but had no family history of coronary artery disease.

**Figure 1:**
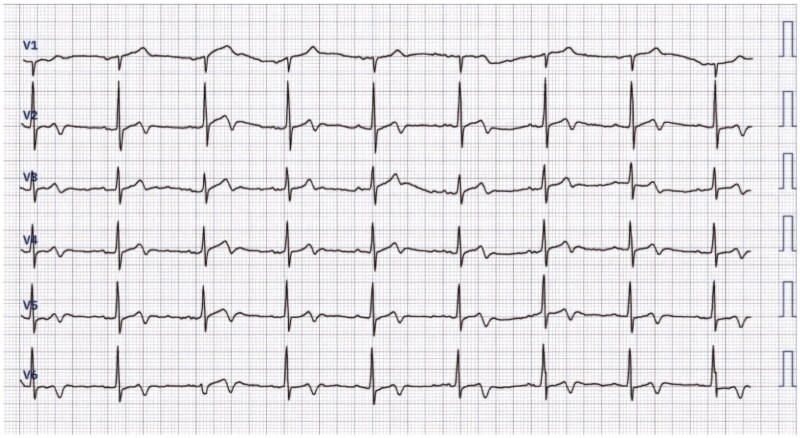
Wellens’ syndrome Type A electrocardiogram pattern.

On examination, vital signs were stable, and no blood pressure asymmetry was noted between arms. ECG showed biphasic T waves in leads V2–V6, consistent with Wellens’ syndrome Type A, which is typically associated with critical LAD stenosis (*Figure 1*). However, transthoracic echocardiography revealed a normal ejection fraction (EF = 65%) with no regional wall motion abnormalities or aortic root pathology.

**Figure 2:**
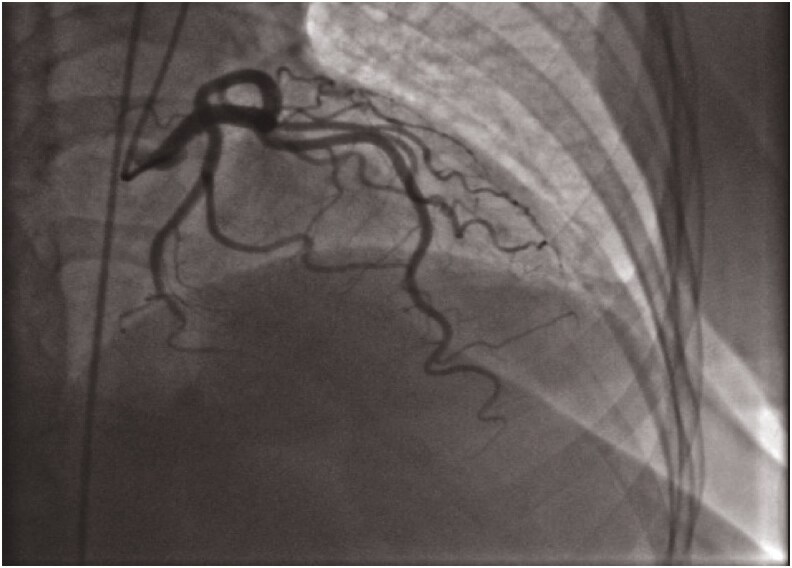
Left anterior oblique caudal view in the coronary angiography showing normal left anterior descending artery.

**Figure 3:**
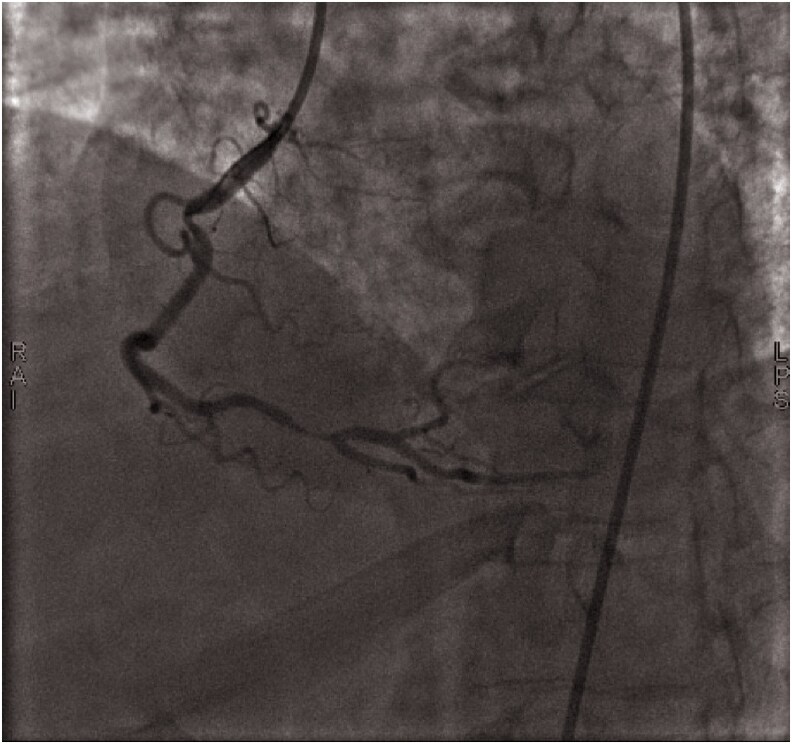
Left anterior oblique cranial view in the coronary angiography showing critical stenosis in the proximal and distal part of right coronary artery.

His troponin level was mildly elevated at 0.1 ng/mL (normal range: 0.02–0.06 ng/mL), raising concerns about ongoing ischaemia. Despite ECG findings suggesting LAD involvement, coronary angiography revealed RCA occlusion (*Figures 2* and *3*). Two drug-eluting stents (DES) were successfully placed in the proximal and distal RCA, restoring TIMI III flow.

## Outcome and follow-up

The patient’s symptoms improved significantly post-procedure. After 48 h of close monitoring in the coronary care unit, he was discharged on antiplatelet and anti-ischaemic therapy, with recommendations for lifestyle modification and improved diabetes management.

## Discussion

His electrocardiographic findings in this case demonstrated biphasic T-wave inversions in the anterior precordial leads, resembling Wellens’ Type A pattern. First described by de Zwaan *et al*. in 1982, Wellens’ syndrome is a crucial marker of proximal LAD stenosis, predicting imminent myocardial infarction.^4^ It is categorized into Type A, which presents with biphasic T waves in V2–V3 (25%), and Type B, characterized by deep, symmetrical T-wave inversions in the precordial leads (75%).^5^ The diagnostic criteria include minimally elevated ST-segments, absent Q waves, T-wave changes in V2–V3, preserved R-wave progression, history of angina, and normal or mildly elevated cardiac markers.^6^

Although classically associated with proximal LAD disease, Wellens-like ECG findings have been observed in RCA stenosis, occurring in approximately 5–10% of cases.^1,7^ In this case, coronary angiography revealed severe RCA stenosis (95% in both proximal and distal segments), suggesting RCA involvement as the primary cause of the ECG pattern. Given the RCA-dominant circulation, transient ischaemia of the apex and anterolateral wall may explain the T-wave abnormalities.

Recently, a shift from the ST elevetion myocardial infarction to the occlusion myocardial infarction (OMI) paradigm has been proposed, emphasizing acute coronary occlusion detection based on ischaemic severity rather than ST-segment elevation.^8^ Within this framework, Wellens’ syndrome is considered an indication for urgent coronary intervention, as it represents a critically stenotic artery at high risk for complete occlusion. The presence of Wellens-like ECG changes in non-LAD territories further supports early angiographic evaluation, even in the absence of ST-elevation.

In addition to transient ischaemia, myocardial oedema has been suggested as a possible mechanism for T-wave inversions in Wellens’ syndrome. Cardiac MRI studies, such as by Migliore *et al*.,^9^ have shown that oedema localized in the anterior myocardial wall can correlate with these electrocardiographic changes. In our case, although cardiac MRI was not performed, the ECG changes resolved within 48 h following revascularization of the RCA, supporting the hypothesis that ischaemia-induced oedema may have contributed to the observed T-wave abnormalities.^9^

This case underscores the importance of recognizing Wellens’ pattern beyond LAD stenosis, particularly in RCA disease. In settings without advanced imaging modalities such as myocardial perfusion imaging or MRI, clinicians must rely on ECG interpretation and angiographic findings for timely intervention. The evolving OMI paradigm reinforces the need for early coronary angiography to prevent myocardial infarction progression.

## Conclusion

This case describes an atypical presentation of Wellens’ syndrome, *in which* biphasic T waves in V2–V6, typically indicating proximal LAD stenosis, were found in a patient with critical RCA occlusion. This highlights the need for coronary angiography, as ECG findings may not always *correspond precisely to* the culprit lesion. The patient underwent successful percutaneous coronary intervention with DES in the proximal and distal RCA, leading to symptom resolution and discharge after 48 h on antiplatelet and anti-ischaemic therapy.

## Data Availability

The data underlying this article are available in the article.

## Lead author biography

Dr Can Baba Arın was born in 1964 and graduated from Çukurova University, Department of Medicine. After completing his specialization in cardiology in 2011 and completing his compulsory service at Van Yüksek Yüksek Hastanesi, he is currently working at Dr. Siyami Ersek Thoracic Cardiovascular Surgery Training and Research Hospital in Istanbul. His areas of interest are invasive cardiology, arrhythmia, echocardiography, and coronary intensive care.
